# The Pro-Tumoral Activity of Heparan Sulfate 3-*O*-Sulfotransferase 3B (HS3ST3B) in Breast Cancer MDA-MB-231 Cells Is Dependent on the Expression of Neuropilin-1

**DOI:** 10.3390/molecules23102718

**Published:** 2018-10-22

**Authors:** Charles Hellec, Mariama Diawara, Mathieu Carpentier, Agnès Denys, Fabrice Allain

**Affiliations:** Unité de Glycobiologie Structurale et Fonctionnelle, UMR 8576 of the Centre National de la Recherche Scientifique, University of Lille, Villeneuve d’Ascq, F-59655 Lille, France; charles.hellec@gmail.com (C.H.); diawaramariama28@yahoo.fr (M.D.); mathieu.carpentier@univ-lille1.fr (M.C.); agnes.denys@univ-lille1.fr (A.D.)

**Keywords:** heparan sulfate, sulfotransferase, neuropilin, cancer

## Abstract

Heparan sulfate 3-*O*-sulfotransferases (HS3STs) catalyze the maturation step of heparan sulfate (HS) 3-*O*-sulfation. This modification is relatively rare. Moreover, only a few biological processes have been described to be influenced by 3-*O*-sulfated HS, and few ligands have been identified so far. Among them, neuropilin-1 (Nrp1) was reported to exhibit tumor-promoting properties by enhancing the action of various growth factors. We recently demonstrated that transient overexpression of HS3ST2, 3B or 4 enhanced the proliferation of breast cancer MDA-MB-231 cells and promote efficient protection against pro-apoptotic stimuli. Hence, we hypothesized that the pro-tumoral activity of these HS3STs could depend on the expression of Nrp1. To test this, MDA-MB-231 cells were stably transfected with a construct encoding HS3ST3B and the expression of Nrp1 was down-regulated by RNA interference. First, we confirmed that stable expression of HS3ST3B effectively increased cell proliferation and viability. Silencing the expression of Nrp1 markedly attenuated the promoting effects of HS3ST3B, while the same treatment had only a moderate effect on the behavior of the parental cells. Altogether, our findings support the idea that the tumor-promoting effects of HS3ST3B could be dependent on the expression of Nrp1 in cancer cells.

## 1. Introduction

Heparan sulfate (HS) modulates the binding of a large number of ligands, resulting in the regulation of a variety of physiological and pathological processes, such as in embryonic development, homeostasis, inflammatory responses, infections, and tumor growth. Binding to HS can have many effects on the ligands, ranging from simple immobilization to induction of conformational changes, stabilization of receptor-ligand complexes or protein oligomerization. The HS-protein interactions are driven at a first level by the overall sulfation of HS motifs, and then by the spatial arrangement of sulfate groups in given sequences [1,2]. The structural diversity in HS is derived from enzymatic modifications of the glycan backbone formed of alternating d-glucuronic acid (GlcUA) and *N*-acetylated d-glucosamine (GlcNAc) units. In the classical model of HS biosynthesis, this precursor is first subject to partial *N*-deacetylation/*N*-sulfation of GlcNAc residues, which provides the pre-requisite substrate needed for subsequent modifications: C5n epimerization of some GlcUA into l-iduronic acid (IdoUA), 2-*O*-sulfation of uronic acid residues, mainly IdoUA, and 6-*O* and/or 3-*O*-sulfation of GlcN residues. These modifications are catalyzed by complex enzymatic machinery, including *N*-deacetylases/*N*-sulfotransferases (NDSTs), C5-epimerase and 2-*O*, 6-*O* and 3-*O*-sulfotransferases (HS2ST, HS6STs, HS3STs). To date, four NDSTs, three HS6STs and seven HS3STs have been characterized in human, the expression of these isozymes being dependent on cell type and tissue environment [2,3]. HS3STs represent the largest family of HS sulfotransferases, and still the reaction of 3-*O*-sulfation is the rarest modification within HS, compared to *N*-, 6-*O*-GlcN and 2-*O*-UA sulfations [4]. HS3ST1 was described as catalysing the addition of a sulfate group to the 3-OH position of a *N*-sulfated GlcN (GlcNS) residue that is linked to a non-sulfated GlcUA residue at the non-reducing side [5,6]. HS3ST5 exhibits broader substrate specificity and transfers a sulfate group to GlcNS linked to GlcUA or IdoUA irrespective of 2-*O*-sulfation [7,8]. Both of these isozymes are involved in the generation of anticoagulant-active HS/heparin sequences, as they participate in the synthesis of the binding motifs for antithrombin-(AT)-III. HS3ST2, 3A, 3B, 4 and 6 transfer a sulfate group to the 3-OH position of GlcNS residue that is linked to an adjacent upstream 2-*O*-sulfated IdoUA. These isozymes, often referred as “gD-type” HS3STs, were reported as providing the functional HS-binding motifs for the protein gD of type I herpes simplex virus (HSV-1) and to assist viral infection [9,10,11,12,13,14].

Apart from their roles in the anti-coagulant properties of HS/heparin and the entry of HSV-1 into host cells, little is known concerning the functions of 3-*O*-sulfated motifs in other biological processes [4]. It is also worth noting that conflicting literature reported that certain HS3STs may act as either anti-oncogenic or tumor-promoting regulators. On the one hand, aberrant methylation of the genes encoding HS3ST2 and HS3ST3A was described in various cancers and tumor cells, and reversing methylation restored their expression and resulted in the suppression of tumor cell growth [15,16,17,18]. On the other hand, HS3ST2, 3B and 4 were reported to promote cancer progression. HS3ST2 overexpression increases the viability and invasiveness of the breast cancer MDA-MB-231 cells [19]. HS3ST3B induces an epithelial-mesenchymal transition in pancreatic cancer cells [20] and promotes the proliferation of acute myeloid leukemia cells [21]. Pathological expression of HS3ST4 plays a deleterious role in the escape of cancer cells from the immune system [22]. In line with these last findings, we recently demonstrated that transient expression of HS3ST2, 3B and 4 enhances the proliferation of MDA-MB-231 cells and promotes efficient protection against cell death, which suggests that these isozymes may display a prominent role in breast cancer expansion [23].

To date, hundreds of HS-binding proteins have been identified, but only a few ligands are known to selectively interact with 3-*O*-sulfated HS motifs [4]. Among them, neuropilin-1 (Nrp1) was recently described as a preferential ligand for HS3ST2-modified HS [24]. Nrp1 is a transmembrane glycoprotein, initially identified in neuronal and endothelial cells as a co-receptor for semaphorins and vascular endothelial growth factors (VEGF). Importantly, it is also expressed in a number of epithelial cancer cells, wherein it contributes to cell growth, migration, invasion and survival by interacting with a large number of growth factors and their cognate signaling receptors [25,26,27,28,29]. Hence, we hypothesized that the functional impact of HS3ST3B expression in MDA-MB-231 cells could be related to the presence of Nrp1. To test this assumption, MDA-MB-231 cells were stably transfected with a construct encoding HS3ST3B and the expression of Nrp1 was knocked down by RNA interference. Altogether, our findings suggest that the tumor-promoting effects of HS3ST3B are dependent on the expression of Nrp1 in cancer cells.

## 2. Results

### 2.1. Expression of HS3ST3B by Stable Transfection in MDA-MB-231 Cells

**Figure 1:**
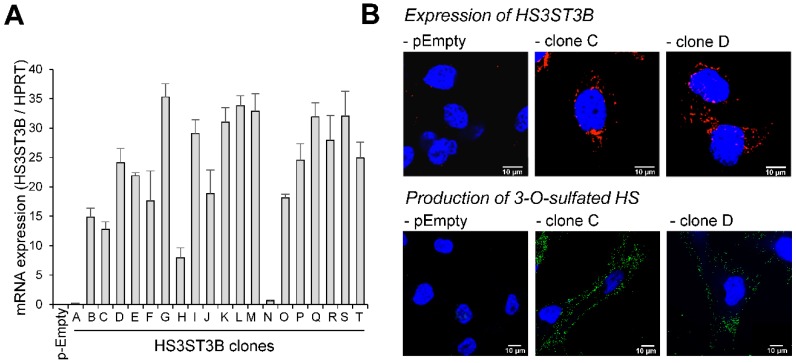
Stable expression of HS3ST3B in MDA-MB-231 cells. Cells were transfected with the expression vector encoding HS3ST3B and then cultured in complete DMEM medium in the presence of 400 µg/mL G418. After 14 days of culture, individual colonies were isolated by limit dilution and amplified in medium supplemented with G418. In parallel, cells were transfected with the empty vector to obtain the control parental cells (pEmpty). (**A**) Following RNA extraction, the mRNA level of HS3ST3B was quantified by real-time RT-PCR in each clone. Relative abundance of the transcripts was normalized to endogenous HPRT mRNA. Data are means ± SD of triplicates. (**B**) HS3ST3B expression in the clones C and D was analyzed by confocal microscopy. To this end, cells were seeded on glass coverslips, permeabilized and then incubated in the presence of anti-HS3ST3B antibodies. After wash, they were immunostained with secondary antibodies conjugated to Alexa-568, in order to highlight the enzyme in red fluorescence. For the detection of 3-*O*-sulfated motifs, recombinant HSV-1 gD (10 μg/mL) was incubated with primary anti-gD antibody for 30 min at 4 °C, and the immune complex was added to cells for an additional 30 min-incubation. After washing, cells were fixed and incubated for 1 h with Alexa 488-conjugated secondary antibody (green fluorescence). In all the microscopy experiments, nuclei were stained in blue with DAPI, in order to visualize cell nuclei (*N* = 3 separate experiments; *n* = 30 cells). Scale bar = 10 µm.

Following transfection with the expression vector encoding human HS3ST3B, twenty individual G418-resistant colonies were isolated by limiting dilution and the expression of HS3ST3B transcripts was analyzed by RT-PCR (Figure 1A). As described in [23], the level of HS3ST3B expression is very low in the parental MDA-MB 231 cells. Strong overexpression was observed in 18 of the resistant clones, with a 160 to 700-fold increase in the levels of mRNA encoding the isozyme. For the next experiments, it was decided to retain the clones C and D, because of a medium level of expression of HS3ST3B (×250 and ×480 respectively).

To confirm that HS3ST3B was efficiently produced in both of these clones, cells were fixed, permeabilized and then incubated with an anti-HS3ST3B antibody. Thereafter, they were incubated with secondary antibody conjugated with Alexa-568. The fluorescence signal was analyzed by confocal microscopy. As shown in Figure 1B, the enzyme could be visualized in both clones, while it was not detectable in the parental cells. We also checked that HS3ST3B was active in stably transfected MDA-MB-231 cells. A recombinant form of HSV-1 gD has already been used to characterize the interaction of the viral glycoprotein with HS3ST-modified HS [8,9,10,11] and to visualize its binding to the surface of cells that had been transfected with constructs encoding HS3ST2 and 3B [30]. Hence, we used here the binding of recombinant HSV-1 gD as a read-out to verify that the stable expression of HS3ST3B resulted in the production of a functional enzyme in MDA-MB-231 cells. As expected, we found that HSV-1 gD binding could be visualized with HS3ST3B expressing cells, while we did not observe any binding of HSV-1 gD to parental cells. (Figure 1B).

### 2.2. Effect of the Stable Expression of HS3ST3B on the Proliferation and Viability of MBA-MB-231 Cells

**Figure 2:**
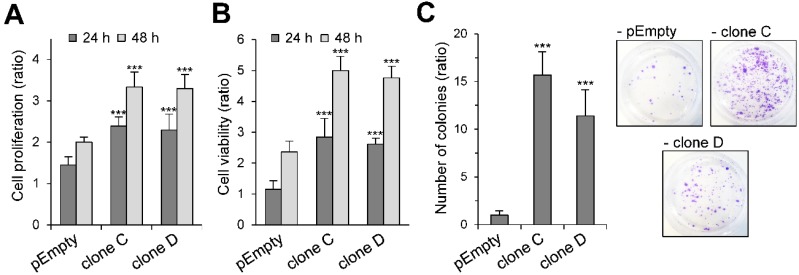
Effect of the stable expression of HS3ST3B on the growth and survival of MDA-MB-231 cells. Parental (pEmpty) and HS3ST3B expressing (clones C and D) cells were cultured with 1% FCS for 24 and 48 h. At each time point, the effect of HS3ST3B expression on the cell growth was estimated by (**A**) cell counting and (**B**) MTS assay. Results are expressed as fold changes by comparison with the cells that have been initially added into the wells. Data are means ± S.D. from three separate experiments performed independently (*** *p* < 0.001, significantly different when compared with the control cells). (**C**) Equal numbers of the parental and HS3ST3B expressing cells were seeded in six well plates (2000 per well) and maintained for nine days in DMEM complemented with 1% FCS, after which time the colonies were stained with crystal violet. The left panel represents the quantification of the colonies per well. Results are expressed as fold changes by comparison with the control cells transfected with empty vector. Data are means ± S.D. from three separate experiments performed independently (*** *p* < 0.001, significantly different when compared with the control cells).

One of the principal hallmarks of cancer cells is their uncontrolled growth, which results in increased proliferation and viability. In previous works, we demonstrated that transient expression of HS3ST3B resulted in a significant increase in the growth of MDA-MB-231 cells [23]. Hence, we tested whether stable expression of the enzyme had similar enhancing effects on cell proliferation and viability. When compared with the MDA-MB-231 cells transfected with an empty plasmid, we found that the rates of proliferation of the clones C and D were similarly increased after 24 h and 48 h of culture in the presence of 1% fetal calf serum (FCS), without any notable difference between both clones (×1.6 as compared with the control cells) (Figure 2A). Similar enhancement in the viability of the HS3ST3B expressing cells was observed. The rates of cell viability had more than doubled at 24 h and 48 h of culture with 1% FCS, as compared with the control cells (Figure 2B). Finally, we analyzed the colony forming capacity of HS3ST3B expressing cells. The ability of individual cancer cells to grow into colonies is indeed a consequence of the activation of survival signals leading to enhanced cellular viability. As shown in Figure 2C, stable transfection with the HS3ST3B expression vector resulted in a more than 10-fold increase in the colony forming capacity of MDA-MB-231 cells, compared to the parental cells. Moreover, no significant difference could be observed between the clones C and D. Altogether, these first results confirmed that the stable expression of HS3ST3B was efficient in enhancing the growth of MDA-MB-231 cells.

### 2.3. Participation of Nrp1 to the Enhancing Effect of HS3ST3B on MDA-MB-231 Cell Growth

**Figure 3:**
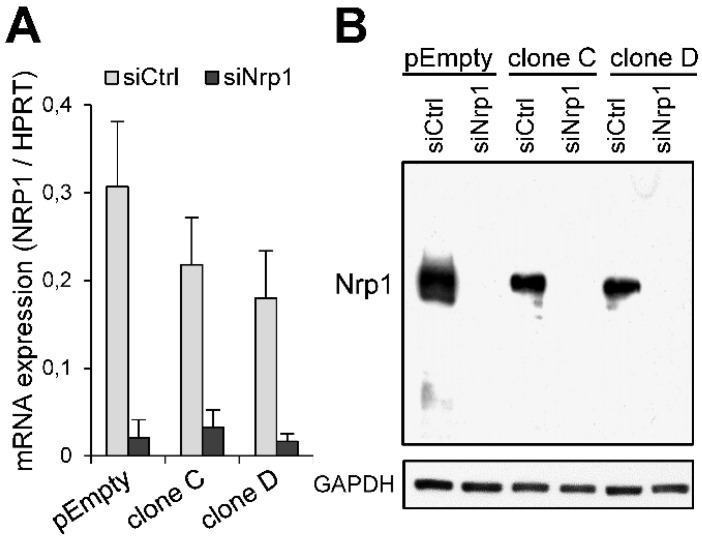
Knockdown of the expression of Nrp1 in MDA-MB-231 cells by RNA interference. Cells were transfected with the negative control siRNA (siCtrl) or specific siRNA targeting Nrp1 (siNrp1). (**A**) After 24 h of treatment, the levels of mRNA encoding Nrp1 were determined by real-time RT-PCR. Relative abundance of the transcripts was normalized to endogenous HPRT mRNA. Data are means ± SD of triplicates. (**B**) The efficacy of specific siRNA to knockdown the expression of Nrp1 was verified by Western blot 48 h post-transfection. Parallel immunoblotting with anti-GAPDH confirmed equal loading of the samples. Data are representative of three independent experiments.

Thacker et al. [24] reported that Nrp1 preferentially interacts with 3-*O*-sulfated HS. Hence, we sought to determine whether the functional impact of HS3ST3B could be linked to the expression of Nrp1 in MDA-MB-231 cells. To decipher the relationships between both molecules, we decided to silence the expression of Nrp1 by RNA interference. Treatment of the parental MDA-MB-231 cells and the clones C and D with a specific siRNA targeting Nrp1 (siNrp1) resulted in a significant decrease in the levels of corresponding mRNA. After 24 h of treatment, the inhibitory effects of siRNA were estimated at more than 90%, compared to the results obtained with the negative control siRNA (siCtrl) (Figure 3A). Moreover, knockdown of the Nrp1 mRNA was accompanied by a loss of the Nrp1 protein, as shown by western blot (Figure 3B), thus validating the siRNA used in this study.

**Figure 4:**
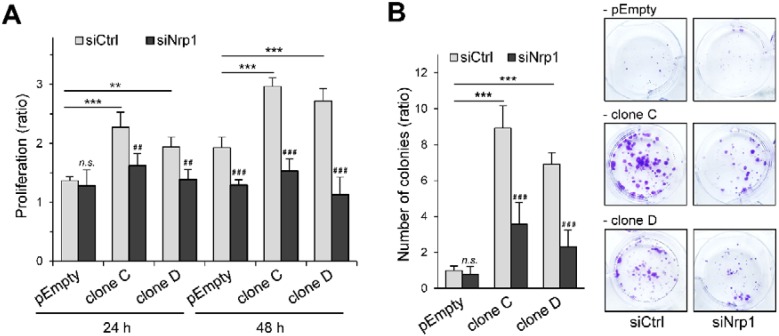
Effect of Nrp1 silencing on HS3ST3B-mediated cell proliferation and survival. Parental (pEmpty) and HS3ST3B expressing (clones C and D) cells were transfected with the siRNA targeting Nrp1 (siNrp1) or negative control siRNA (siCtrl) and then cultured for 24 h in complete medium. (**A**) After wash, cells were collected and cultured in medium containing 1% FCS for 24 h and 48 h. At each time, the effect of HS3ST3B on the proliferation of MDA-MB-231 cells was estimated by cell counting. Results are expressed as fold changes by comparison with the cells that have been initially added into the wells. Data are means ± S.D. from three separate experiments performed independently (** *p* < 0.01, *** *p* < 0.001, significantly different when compared with the parental cells; ^##^ *p* < 0.01, ^###^ *p* < 0.001, significantly different when compared with the siCtrl-treated cells; n.s. not significantly different). (**B**) Cells were seeded in six well plates (2000 per well) and cultured for nine days in the presence of 1% FCS, after which the colonies were stained with crystal violet. The left panel represents the quantification of the colonies per well. Results are expressed as fold changes by comparison with the parental cells that have been transfected with siCtrl. Data are means ± S.D. from three separate experiments performed independently (*** *p* < 0.001, significantly different when compared with the parental cells; ^###^ *p* < 0.001, significantly different when compared with the siCtrl-treated cells; n.s. not significantly different).

We next examined the effect of HS3ST3B on the proliferation rates of siRNA-treated cells (Figure 4A). As expected, cell treatment with siCtrl had no inhibitory effect on the functional impact of HS3ST3B expression. The proliferation rates of the clones C and D were indeed increased after 24 h and 48 h of culture in the presence of 1% FCS, compared with the parental cells. In contrast, silencing the expression of Nrp1 significantly attenuated the promoting effect induced by HS3ST3B expression.

Interestingly, we did not observe any notable difference in the cell proliferation rates between the parental and HS3ST3B expressing cells that have been treated with siNrp1, suggesting that silencing the expression of Nrp1 reversed the advantage given by HS3ST3B expression on cell proliferation. Then, we analyzed the effect of siRNA treatment on the colony forming capacity of MDA-MB-231 cells (Figure 4B). We confirmed that HS3ST3B-mediated increase in cell viability was not altered by treatment with the siRNA control. Indeed, HS3ST3B expression resulted in more than an 8-fold increase in the colony forming capacity of the clones C and D, compared with the parental cells. In contrast, silencing the expression of Nrp1 resulted in a dramatic loss of viability in HS3ST3B expressing cells, which indicates that the absence of Nrp1 also altered the enhancing effect of HS3ST3B on cell survival.

### 2.4. Participation of Nrp1 to HS3ST3B-Mediated Protection against Cell Death

**Figure 5:**
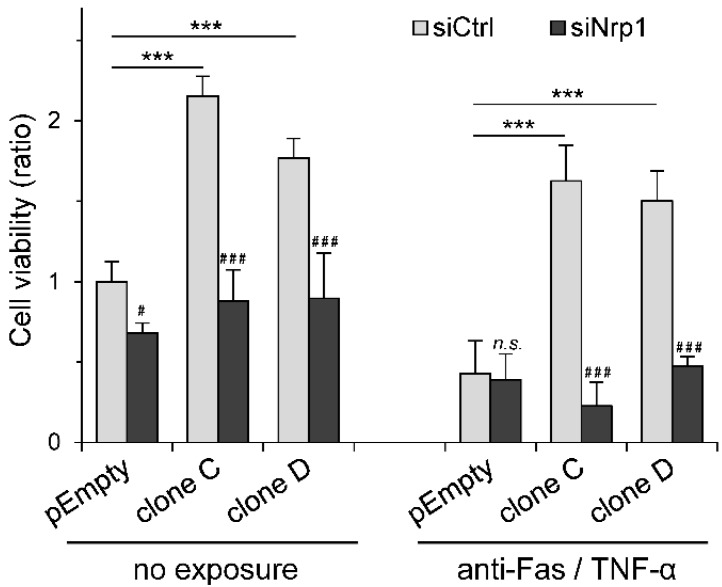
Effect of Nrp1 silencing on HS3ST3B-mediated protection against apoptosis. Parental (pEmpty) and HS3ST3B expressing (clones C and D) cells were transfected with siNrp1 or siCtrl and then cultured for 24 h in complete medium. After wash, cells were collected and cultured in medium containing 1% FCS in the absence (control) or presence of a mixture of anti-Fas antibody (100 ng/mL) and TNF-α (100 ng/mL) for 24 h. Thereafter, the number of viable cells was estimated by using MTS assay. Results are expressed as fold changes by comparison with the parental cells that have been transfected with siCtrl and cultured in the absence of pro-apoptotic stimulus. Data correspond to means ± S.D. from three independent experiments (*** *p* < 0.001, significantly different when compared with the parental cells; ^#^ *p* < 0.05, ^###^ *p* < 0.001, significantly different when compared with the siCtrl-treated cells; n.s., not significant).

We previously shown that transient expression of HS3ST3B was effective in reducing cell death in MDA-MB-231 cells exposed to pro-apoptotic stimuli [23]. To determine whether this protective effect could be related to the expression of Nrp1, HS3ST3B-transfected cells were treated with either siCtrl or specific siNrp1 and then exposed to a mixture containing an anti-Fas antibody and TNF-α (Figure 5).

We used these reagents in order to mimic the delivery of extracellular death signals mediated by a TNF receptor and the Fas antigen [31]. In the first experiment, cell viability was analyzed without the pro-apoptotic treatment. As expected, we confirmed that HS3ST3B-mediated increase in cell viability was not altered by treatment with the siRNA control. We found indeed that the stable expression of HS3ST3B resulted in a 2-fold increase in cell viability of the clones C and D, compared with the parental cells. In contrast, silencing the expression of Nrp1 strongly reduced the enhancing effect induced by HS3ST3B, while it had only a minor effect on the viability of the parental cells. Moreover, no notable difference in the rates of cell viability could be observed between the parental and HS3ST3B expressing cells that have been treated with siNrp1 (Figure 5), which further supports the idea that silencing Nrp1 altered the functional impact of HS3ST3B expression in MDA-MB-231 cells. In the next experiment, cells were treated with a mixture of anti-Fas/TNF-α in order to induce cell death. As expected, we found that exposure of the parental cells to the pro-apoptotic stimuli reduced the rate of cell viability by more than half, regardless of treatment with either siCtrl or siNrp1. In contrast, the expression of HS3ST3B efficiently attenuated the loss of cell viability induced by the pro-apoptotic stimuli in siCtrl-treated cells. When compared with the non-exposed cells, we found that the rates of cell viability were only reduced by 25% and 15% in the clones C and D respectively, indicating that HS3ST3B was effective for protecting MDA-MB-231 cells against apoptosis. Conversely, silencing the expression of Nrp1 in HS3ST3B expressing cells resulted in a dramatic loss of viability down to a level comparable to those in the parental cells after exposure to the mixture of anti-Fas/TNF-α (Figure 5). Thus, these last results indicate that the absence of Nrp1 abolished the protective effect of HS3ST3B on MDA-MB-231 cells against apoptotic stimuli.

### 2.5. Role of Nrp1 in the Activation of Akt and Src in HS3ST3B Expressing Cells

**Figure 6:**
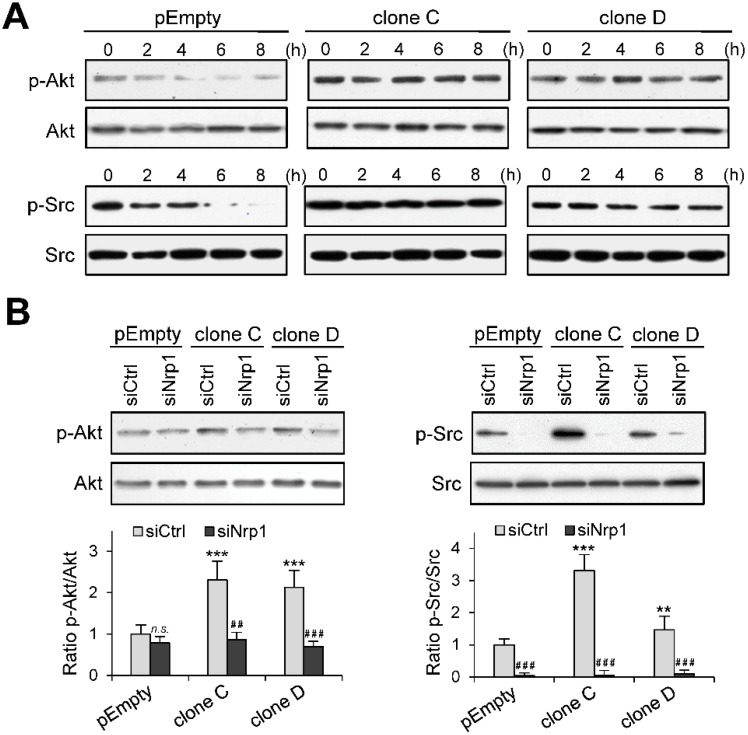
Effect of Nrp1 silencing on HS3ST3B-mediated activation of Akt and Src kinases. Parental (pEmpty) and HS3ST3B expressing (clones C and D) cells were transfected with siRNA targeting Nrp1 (siNrp1) or negative siRNA (siCtrl) and then cultured for 24 h in complete medium. (**A**) After wash, cells were cultured in the absence of FCS for 8 h. Every two h, cells were collected and lysed. Proteins were then separated by SDS-PAGE and subjected to Western blotting with antibodies to the phosphorylated forms of Akt and Src. Parallel immunoblotting with antibodies to Akt and Src regardless of their phosphorylation status confirmed equal loading of samples. Representative results from three independent experiments are shown. (**B**) Parental and HS3ST3B expressing (clones C and D) cells were transfected with siRNAs as above. After serum starvation for 2 h, the phosphorylated forms of Akt and Src was detected in cell lysates by Western blot. Parallel immunoblotting with anti-Akt and anti-Src antibodies confirmed equal loading of the samples. Representative results from three independent experiments are shown. Histograms represent the quantification of the phosphorylation status of Akt and Src. Data were normalized to the parental cells that have been transfected with siCtrl (** *p* < 0.01, *** *p* < 0.001, significantly different when compared with the parental cells; ^##^ *p* < 0.01, ^###^ *p* < 0.001, significantly different when compared with the siCtrl-treated cells; n.s., not significant).

Sustained activation of Akt and Src has been associated with an increase in tumor cell growth and enhancement of pro-survival signals [32,33]. To explore the functional impact of HS3ST3B expression on the activation of these kinases, the parental MDA-MB-231 cells and the clones C and D were cultured in the absence of serum for 8 h. In this time-course experiment, cells were collected every 2 h and the phosphorylation status of Akt and Src was analyzed by western blot. The levels of phosphorylated forms of both Akt and Src decreased gradually over time in the parental cells (Figure 6A). These results were as expected given the deprivation of mitogen stimulation after serum starvation. In contrast, the activation of Akt and Src in HS3ST3B expressing cells was not modified throughout the experiments, as demonstrated by the sustained and persistent phosphorylation of both kinases (Figure 6A). These results suggest that the stable expression of HS3ST3B probably favored the activation of an autocrine mechanism, which maintained long-term activation of intracellular signaling pathways even in the absence of external stimuli.

Then, we sought confirmation whether Nrp1 was involved in increased activation of Akt and Src in HS3ST3B expressing cells. To this end, cells were pre-treated with siRNAs and then serum-starved for 2 h, after which time the phosphorylation status of both kinases was analyzed by Western blot. Silencing Nrp1 in the parental cells had no significant effect on the phosphorylation level of Akt (Figure 6B), indicating that the basal activation of the kinase is likely not dependent on Nrp1. In contrast, the same treatment efficiently reduced the activation of Akt in HS3ST3B expressing cells. The remnant level of Akt phosphorylation was similar to that observed in the parental cells, which suggests that the enhancing effect of HS3ST3B on the activation of Akt relied upon the expression of Nrp1. Unlike Akt, the Src activation appeared dependent on the expression of Nrp1. Indeed, the loss of Nrp1 in the parental cells resulted in a strong reduction in the phosphorylation level of Src (Figure 6B). A similar reduction was observed in the clones C and D, indicating that Nrp1 is also necessary to support the HS3ST3B-mediated activation of Src.

## 3. Discussion

In normal physiological conditions, Nrp1 is expressed by a variety of cell types, including endothelial cells, neurons, macrophages and some T cell subsets. It was first identified as a receptor for the class 3 semaphorins, which are involved in axonal guidance in embryonic development. Subsequently, it was found to interact with several members of the VEGF family and to promote angiogenesis. In view of this, Nrp1 was considered as a co-receptor for semaphorin-3 and VEGF family members [26,27,28]. Interestingly, heparin was reported to markedly increase the affinity of VEGF for Nrp1 and to contribute to the formation of a complex incorporating Nrp1, VEGF and its receptors [34,35]. Consistent with this observation, Thacker et al. [24] recently demonstrated that Nrp1 interacts preferentially with 3-*O*-sulfated HS. They used a classical approach based on affinity chromatography and salt elution. To this end, HS lacking 3-*O*-sulfate groups was modified by recombinant HS3ST2 and coupled to chromatography resin. Then, they validated the importance of 3-*O*-sulfation in the functions of Nrp1 in binding assays and by showing that Nrp1-dependent axonal growth cone collapse was dependent on the expression of HS3ST2. They also demonstrated that 3-*O*-sulfated HS were more potent than HS chains lacking 3-*O*-sulfate groups in the inhibition of VEGF-mediated endothelial sprouting. Thus, it can be concluded from their results that 3-*O*-sulfation resulted in the generation of biologically active motifs for Nrp1. In addition to semaphorin-3 and VEGF family members, Nrp1 was described as a co-receptor for a broad spectrum of ligands, including transforming growth factor β1 (TGF-β1), hepatocyte growth factor (HGF), platelet derived growth factor (PDGF), epidermal growth factor (EGF) and their respective signaling receptors. Moreover, accumulating evidence has associated Nrp1 expression in cancer cells with tumor progression [25,26,27,28,29]. High levels of Nrp1 have been associated with poor outcome in patients with cancers of various origins and correlated with invasive behavior and metastatic potential. On the other hand, Nrp1 expression was found upregulated in epithelial cells upon neoplastic transformation [36]. These observations suggested that Nrp1 overexpression in cancer cells may lead to the acquisition of a functional advantage at the cellular level. Notably, silencing Nrp1 expression in carcinoma cells was shown to impair proliferation, survival and invasion in vitro, while Nrp1 overexpression can inhibit cancer cell apoptosis. These effects have been explained by the role of Nrp1 in supporting the activation of signaling pathways elicited by a variety of growth factors, such as VEGF, EGF, HGF and PDGF [26,27,36,37,38].

We demonstrated here that MDA-MB-231 cells carrying HS3ST3B expression displayed a significant increase in cell proliferation and survival in culture. Complementary to this, we found that the stable expression of HS3ST3B resulted in sustained and persistent activation of Akt and Src in serum-starved cells, suggesting an autocrine mechanism of cell activation. Such an aberrant activation of Akt and Src has been reported to favor tumor growth and to enhance the resistance to apoptosis [32,33]. We have reproduced the same experiments with cells into which the expression of Nrp1 was knocked-down by RNA interference. We found that silencing the expression of Nrp1 strongly reduced the proliferation and viability of HS3ST3B expressing cells, while the same treatment had only a moderate effect on the parental cells. Moreover, Nrp1 knockdown resulted in a dramatic loss of the resistance of HS3ST3B expressing cells exposed to pro-apoptotic stimuli, which means that the absence of Nrp1 abolished the protective effect of HS3ST3B. Finally, we found that the activation of Akt and Src was reduced in HS3ST3B expressing cells to a similar level to that observed in the parental cells upon Nrp1 silencing. Altogether, these results suggest that the tumor-promoting effects of HS3ST3B could be related to the role of Nrp1 in the formation of signaling complexes with growth factors and their cognate receptors.

In endothelial cells, HS was shown to regulate VEGF activity not only by binding VEGF directly but also by interacting with its signaling receptors and Nrp1. Thus, much work has been done to understand the role of HS in the formation of signaling complexes [39]. Specifically, Teran and Nugent [35] proposed that Nrp1, VEGF and its receptors act synergistically with cell surface HS to form high-affinity signaling complexes. They showed that the ability of Nrp1 to influence VEGF activity was eliminated by chlorate treatment. This inhibition could be rescued by the addition of heparin, which supports the model whereby the interactions between Nrp1, VEGF and its signaling receptors are dependent on the presence of HS. Thus, it was proposed that HS could act by facilitating the interactions between Nrp1, VEGF and its receptors and/or by stabilizing the signaling complexes [35]. Taking these findings together, it is tempting to hypothesize that HS3ST3B-modified HS could act by enhancing the interactions between Nrp1, growth factors and their receptors in cancer cells. Accordingly, 3-*O*-sulfated HS might facilitate the formation of the signaling complexes, and/or reinforce the interactions between Nrp1 and its binding partners. Further investigations are currently in progress to decipher the molecular interactions between HS3ST3B-modified HS, Nrp1 and growth factors in MDA-MB-231 cells.

In our previous works, we reported that overexpression of HS3ST3B in MDA-MB-231 cells resulted in the production of HS containing 3-*O*-sulfated disaccharides [23]. Among them, the tetrasulfated HexUA2S-GlcNS3S6S unit was already described as a major product of HS3ST3 isozymes [40]. Because the binding properties of HS/heparin are primarily driven by the interactions between positively charged amino acid residues in protein ligands and negatively charged sulfate groups in HS sequences, it could be argued that increase in HS 3-*O*-sulfation may lead to non-specific interaction. Nevertheless, we did not observe any increase in the overall sulfation level of HS purified from HS3ST3B expressing cells, as compared with the parental cells [23]. On the other hand, 3-*O*-sulfation can have a profound biological effect without inducing high affinity binding sites for the protein ligands. Indeed, ectopic expression of HS3STs in CHO cells, which are normally resistant to HSV-1 infection, resulted in a strong increase in the susceptibility to viral infection, even though the affinity of HSV-1 gD for 3-*O*-sulfated HS is relatively low (~2 μM) [4,9,10,11,12,13,14].

In the literature, the reaction of 3-*O*-sulfation has often been considered as the last modification in HS biosynthesis. This statement arises from the observations that: 3-*O*-sulfation is a rare modification compared to *N*-, 6-*O*- and 2-*O*-sulfations; HS3STs can produce a tetrasulfated disaccharide as a major product from HS/heparin; the acceptor substrates are already modified at other positions by NDST, HS2ST and C5 epimerase. In this scenario, the reaction of 6-*O*-sulfation is thought to be dispensable [3,40]. In their recent work, Wang et al. [41] reexamined the substrate specificity of HS3ST1 and HS3ST3. They found that HS3ST1 preferentially modifies 6-*O*-sulfated disaccharide units. Thus, for the synthesis of oligosaccharides containing the GlcA-GlcNS3S6S disaccharide unit, the 3-*O*-sulfation by HS3ST1 has to be performed after the 6-*O*-sulfation step. In contrast, they found that HS3ST3 exhibits a preferential activity towards oligosaccharides without 6-*O*-sulfation. However, the tetrasulfated IdoUA2S-GlcNS3S6S unit was described as one of the major product of HS3ST3 [40]. Hence, the substrate requirements of HS3ST3 imply that 3-*O*-sulfation reaction occurs prior to the 6-*O*-sulfation step in the synthesis of this disaccharide unit. These findings raise the possibility that 3-*O*-sulfated motifs might be synthesized through different pathways [41].

There is conflicting evidence in the literature regarding the role of HS3STs in cancer. Hyper-methylation of the genes encoding HS3ST2 and HS3ST3A was described in a number of cancer cells. Reversing methylation restored the expression of theses isozymes and resulted in the suppression of tumor cell growth, suggesting anti-oncogenic properties [15,16,17,18]. Conversely, we and others demonstrated that forced expression of HS3ST2 resulted in increased proliferation and viability of MDA-MB-231 cells [19,23]. We also showed that HS3ST3B and HS3ST4 shared with HS3ST2 the same promoting effects, which revealed that the expression of these isozymes had similar functional impact on cancer cell behavior. Hence, these observations suggested that the loss of expression of one HS3ST could be compensated with the expression of another one, depending on the molecular signature of cancer cells and on tumor environment. Notably, upregulation of the expression of HS3ST3B has been observed in many cell types exposed to inflammatory and immune stimuli [42,43,44,45]. During cancer progression, developing tumor cells are exposed to pro-inflammatory and immunomodulatory cytokines that enhance immune anti-tumoral responses. In order to evade this immune pressure, tumor cells change their intrinsic features and modify their microenvironment, which result in the emergence of cellular variants with a less immunogenic phenotype and aberrant activation of oncogenic pathways that endows them with enhanced properties to survive and proliferate [46]. Accordingly, these findings raise the hypothesis that increasing the expression of HS3ST3B in cancer cells could be a potential escape mechanism that reduces the anti-tumoral pressure exerted by the immune system. This notion is in agreement with recent data showing high expression of HS3ST3B in non-small cell lung cancer biopsies, while it was weakly expressed in matched normal tissues [47].

In conclusion, we demonstrated that HS3ST3B enhances the proliferation and survival of breast cancer MDA-MB-231 cells, via a mechanism that is dependent on Nrp1. These findings reveal a new pathway that links 3-*O*-sulfated HS and Nrp1 in cancer expansion, and highlight the clinical value of HS3ST3B as a future target for therapeutic approaches.

## 4. Materials and Methods

### 4.1. Materials

Antibodies to human HS3ST3B and Nrp1 were purchased from R&D Systems (Minneapolis, MN, USA). Antibodies to phospho-Akt(S^473^), total Akt, phospho-Src(Y^416^), total c-Src, and secondary antibodies conjugated to HRP were from Cell Signaling Technology (Danvers, MA, USA). Secondary antibodies conjugated to Alexa-488 (green fluorescent dye) and to Alexa-568 (red fluorescent dye) were from Thermo Fisher Scientific (Waltham, MA, USA). Recombinant HSV-1 gD protein and antibody to HSV-1 gD (clone 1.3) were obtained from Antibodies-online (Aachen, Germany) and Abcam (Cambridge, UK), respectively. Antibody to GAPDH was from Santa Cruz (Santa Cruz, CA, USA). Other chemicals were from Sigma-Aldrich (Darmstadt, Germany) unless otherwise specified.

### 4.2. Cell Culture and Transfection

Human breast cancer MDA-MB-231 cells (ATCC^®^ HTB-26™) were routinely cultured at 37 °C in Dulbecco’s Modified Eagle Medium (DMEM) supplemented with 10% FCS (Lonza, Verviers, Belgium), in an atmosphere containing 5% CO_2_. Construction of the plasmid encoding human HS3ST3B has been described in [23]. Transfection was performed with Lipofectamine^®^ 2000, according to the manufacturer’s instructions (Thermo Fisher), after which cells were cultured in culture medium in the presence of 400 µg/mL G418 (Invitrogen, Carlsbad, CA, USA). After 14 days of culture, G418-resistant colonies were isolated by limiting dilution and then amplified in culture medium supplemented with 400 µg/mL G418. In parallel, MDA-MB-231 cells were transfected with the empty pcDNA3.1 vector (Thermo Fisher) to obtain the control parental cells.

### 4.3. Measurement of Cell Proliferation and Viability

Cells were plated at 5 × 10^4^ cells/mL in 4 mL of DMEM supplemented with 1% FCS and cultured for 24 h or 48 h. At each time, cells were collected and counted with Trypan Blue to exclude dead cells. In parallel, cell viability was analyzed by using the Cell-Titer 96 Aqueous Non-Radioactive Cell Proliferation Assay kit (Promega, Fitchburg, MA, USA), as described in [23]. Briefly, 2 × 10^3^ cells were plated in 200 µL culture medium. After 24 or 48 h of culture, 20 µL of the MTS/PMS (95:5, *v*/*v*) solution was added to each well and the reaction was developed at 37 °C for 1 h. Absorbance was measured at 490 nm using a BioTek Epoch microplate reader (BioTek Instruments, Winooski, VT, USA). In some experiments, cell apoptosis was induced by the addition of 100 ng/mL of anti-Fas antibody (Merck, Darmstadt, Germany) and 100 ng/mL TNF-α (PeproTech, Rocky Hill, CT, USA) for 24 h. Thereafter, the number of remaining viable cells was estimated by using the MTS/PMS reagents.

### 4.4. Colony Formation Assay

Cells (2 × 10^3^ per point) were seeded in six-well plates and cultured in the presence of 1% FCS for 9 days. After wash with phosphate buffer saline (PBS), the colonies were fixed with 4% paraformaldehyde in 0.1 M sodium phosphate buffer (pH 7.2) for 30 min, and then stained with 0.05% crystal violet for 15 min, as described in [23]. After extensive wash with water, the number of colonies was estimated by measuring the amount of dye (absorbance at 570 nm) released from the cells after treatment with methanol.

### 4.5. RNA Interference

A synthetic small-interfering RNA (siRNA) duplex corresponding to the sequence of the Nrp1 mRNA (NM_003873) 5′-GGUCCUGAAUGUUCCCAGA-3′ (siNrp1) was designed by Sigma-Aldrich. A control siRNA duplex (MISSION^®^, Sigma-Aldrich) was used as negative control (siCtrl). For silencing experiments, cells were plated at 2 × 10^5^ cells per well (2.5 mL) in DMEM supplemented with 1% FCS and then transfected with siRNA (150 pmoles per well) using Lipofectamine^®^ RNAiMAX (Thermo Fisher), according to the manufacturer’s recommendations.

### 4.6. RNA Isolation and Real-Time RT-PCR

Total RNA was isolated from 2 × 10^5^ cells using the NucleoSpin RNA II kit (Macherey-Nagel, Düren, Germany). Reverse transcription was performed from 1 μg of total RNA by using the Maxima First Strand cDNA Synthesis Kit for RT-qPCR (Thermo Fisher Scientific). Synthetic primers for HS3ST3B and Nrp1 were described in [43,48], respectively. They were checked for their specificity by semi-quantitative RT-PCR on a 2.5% (*w*/*v*) agarose gel. They amplified only one fragment of expected size, for which the sequence was confirmed (GATC Biotech, Constance, Germany). Real-time PCR amplifications were performed using an Mx3000P Multiplex Quantitative PCR system (Agilent Technologies, Santa Clara, CA, USA), as described in [49]. The transcript of HPRT was used as a control to normalize the expression of our genes of interest. The amplification efficiency of each primer pair was performed on serial dilutions of cDNA. The average Ct of triplicate samples was used for analysis.

### 4.7. Immunofluorescence Staining and Analysis

Microscopy experiments were performed essentially as described in [30]. For the detection of HS3ST3B, cells were seeded on glass coverslips, washed with PBS and fixed in 4% paraformaldehyde in PBS for 30 min. They were then permeabilized with 0.1% saponin in PBS, treated with a blocking solution containing 0.2% gelatin, 2% BSA and 2% FCS in PBS and incubated with the anti-HS3ST3B antibody (1:100) in the blocking buffer for 1 h After washing, cells were incubated for 1 h with Alexa 568-conjugated secondary antibodies (1:600) in blocking buffer. For the detection of 3-*O*-sulfated HS, recombinant HSV-1 gD (10 μg/mL) was first incubated with anti-HSV-1 gD antibody (1:100) in blocking buffer for 30 min at 4 °C. The complex was then incubated with the cells for 30 min at room temperature. After washing, cells were fixed and incubated with Alexa 488-conjugated secondary antibody, as above. In all experiments, cells were stained with 500 ng/mL DAPI (Sigma-Aldrich) for 10 min, in order to visualize cell nuclei. Immunofluorescence was analyzed with an inverted Zeiss LSM 780 microscope (Oberkochen, Germany) equipped with a 63 × oil immersion lens. Data were collected using the Zeiss Zen Pro 2.1 software and processed with Image J software (Bethesda, MD, USA).

### 4.8. SDS-PAGE and Western Blot

Cells (4 × 10^5^ per point) were lysed in 150 µL of lysis buffer (50 mM Tris-HCl, 150 mM NaCl, 1% Triton X-100, 0.1% SDS, pH 8.0) supplemented with protease and phosphatase inhibitors (Roche Diagnostics, Meylan, France) for 3 h at 4 °C. Lysates were then clarified by centrifugation and protein content of the supernatants was estimated using micro-BCA protein assay kit (Thermo Fisher Scientific). Samples corresponding to 20 µg of proteins were mixed with Laemmli buffer and boiled for 10 min, after which proteins were separated by SDS-PAGE and transferred onto nitrocellulose (Amersham, Uppsala, Sweden). The membrane was blocked for 1 h in 20 mM Tris buffer (TBS) with 0.05% (*v*/*v*) Tween-20 and 5% (*w*/*v*) BSA (Roche), and then probed with primary antibodies overnight at 4 °C in TBS supplemented with 3% (*w*/*v*) BSA. After washing, HRP-conjugated secondary antibodies (1:10,000) were added for 1 h and immunoreactive proteins were detected using ECL prime Western blotting detection (GE Healthcare). Quantification of immunostaining intensity was performed by using Image J software.

### 4.9. Statistical Analysis

Results are representative of at least three independent experiments conducted with distinct cellular preparations. All values are expressed as the means ± SD. Statistical significance between the different values was analyzed by using one-way ANOVA and two-tailed Student’s *t*-tests, with a threshold of *p* < 0.05 considered as significant.
